# Gain and loss of function of P2X_7_ receptors: mechanisms, pharmacology and relevance to diabetic neuropathic pain

**DOI:** 10.1186/1744-8069-10-37

**Published:** 2014-06-16

**Authors:** Daniel Ursu, Philip Ebert, Emily Langron, Cara Ruble, Leanne Munsie, Wei Zou, Bonnie Fijal, Yue-Wei Qian, Terry A McNearney, Adrian Mogg, Olivera Grubisha, Kalpana Merchant, Emanuele Sher

**Affiliations:** 1 Lilly Research Centre, Eli Lilly & Co. Ltd., Sunninghill Road, GU20 6PH Windlesham, Surrey, UK; 2 Lilly Corporate Center, Eli Lilly and Company, 46285 Indianapolis, Indiana, USA; 3 InVentiv Clinical Health, LLC, 504 Carnegie Center, 08540 Princeton, NJ, USA

**Keywords:** P2X receptors, Single nucleotide polymorphism, Gain-of-function, Pain

## Abstract

**Background:**

Genetic causes of exaggerated or reduced pain sensitivity in humans are well known. Recently, single nucleotide polymorphisms (SNPs) in the gene *P2RX7*, coding for the ATP-gated ion channel P2X_7,_ have been described that cause gain-of-function (GOF) and loss-of-function (LOF), respectively of this channel. Importantly, *P2RX7* SNPs have been associated with more or less severe pain scores in patient suffering of post-mastectomy pain and osteoarthritis.

**Results:**

The functional consequences of some *P2RX7* SNPs (rs208294 (His155Tyr), rs1718119 (Ala348Thr) and rs3751143 (Glu496Ala)) were studied in recombinant cells in vitro. Our findings suggest a correlation between GOF and LOF of P2X_7_ and actual channel protein expression. Both channel and pore function for these mutant P2X_7_ receptors changed in parallel to protein levels. On the other hand, the mutant receptors did not differ in their sensitivity to known P2X_7_ agonists and antagonists. We further demonstrated that in patients with diabetic peripheral neuropathic pain (DPNP), the presence of the GOF SNPs rs208294 (His155Tyr) and rs1718119 (Ala348Thr) is associated, in females, with higher pain intensity scores.

**Conclusions:**

Our present results confirm the physiological relevance of some of the SNPs in the *P2RX7* gene and show that the presence of these genetic variants correlates with pain sensitivity also in a diabetic neuropathic pain patient population.

## Background

The P2X_7_ receptor, coded by the *P2RX7* gene, plays a critical role in mediating disparate physiological functions of extracellular ATP, including the regulation of immune responses, inflammation, bone metabolism, cell proliferation and cancer, as well as neuronal-glial cross-talk in both the peripheral and the central nervous systems [1]. More specifically, in recent years, strong evidence has been accumulating on the involvement of P2X_7_ receptors in various pathological neurological conditions, including inflammatory and neuropathic pain, neuroinflammation, and neurodegeneration [2-6].

P2X_7_ is a member of a family of cationic channels (P2X_1_-P2X_7_), having a homo- or hetero-trimeric stoichiometry [7]. Each of the three subunits contains two trans-membrane domains (TM1 and TM2), a large extracellular loop, and intracellular N and C termini. The seven subunits comprising the P2XR family share approximately 30-40% homology in their primary sequence, but differ vastly in the length of their carboxy termini [1,8]. Several splice variants and SNPs are known for these subunits [9]. The whole P2X family is increasingly recognized as an important opportunity for novel drug discovery [10].

P2X_7_ receptors are activated by relatively high ATP concentrations, in the mM range, normally achieved only in the vicinity of damaged cells, in synaptic clefts, or in the context of paracrine-like cell-cell interactions. A typical, albeit not unique, property of P2X_7_ receptors is the ability, upon prolonged activation by ATP, to transition from a channel function, which allows the passage of only small ions such as Ca^2+^ or K^+^, to a pore function, which allows the passage of larger molecules, up to ~ 900 Da. This transition to a pore function, which can be mediated by protein-protein interactions between P2X_7_ and pannexin-1 subunits [11], as well as changes in permeability of P2X_7_ itself [12], triggers a series of intracellular events and, in particular, maturation of the “inflammasome” [13]. As a consequence, activation of P2X_7_ receptors has been shown to trigger the maturation and/or release of very important inflammatory mediators, most notably IL1-β, TNFα and PGE2 [14-20].

*P2RX7* is known to be a highly polymorphic gene [9]. *P2RX7* SNPs have been recently studied also in the context of nociception. An association was found between specific SNPs in the *P2RX7* gene and pain sensitivity in both mice and humans [21]. Significant variability in allodynia scores was found by analysing a large number of mouse strains subjected to the Spared Nerve Injury (SNI) model. Genetic analysis revealed that the haplotype block with the strongest correlation genome wide was within the *P2RX7* gene. Further experimental work in mice demonstrated that a loss-of-function (LOF) of the mouse P2X_7_ receptor, specifically in its pore function, was responsible for the relative insensitivity in nociceptive testing. Similarly, pain sensitivity was linked to *P2RX7* gene polymorphisms in women with post-mastectomy pain (PMP) and osteoarthritis (OA), with those carrying the gain-of-function (GOF) Tyr155 allele at rs208294 (H155Y) [22] reporting more pain than carriers of the His155 allele. Carriers of the LOF His270 allele at rs7958311 (R270H), reported less pain intensity than carriers of the Arg270 allele.

In this paper we present additional functional studies of the rs208294 (H155Y) GOF variant, and extend these studies to include two additional reported SNPs of interest, the GOF rs1718119 (Ala348Thr) [23] and the LOF rs3751143 (Glu496Ala) [24]. We also generated two additional P2X_7_ expressing clones containing double mutations where the Ala348Thr and Glu496Ala changes were added to the Tyr155 SNP background. Our data support the idea that the functional consequences of some of these SNPs are related to changes in P2X_7_ cellular expression levels, with the receptors expressed maintaining similar function and pharmacology. Furthermore, we share preliminary findings of an association between GOF P2X_7_ SNP variants and reported pain intensity scores in a cohort of patients with diabetic peripheral neuropathic pain (DPNP).

## Results

### *P2RX7* SNPs cause gain- and loss-of function phenotypes

**Figure 1:**
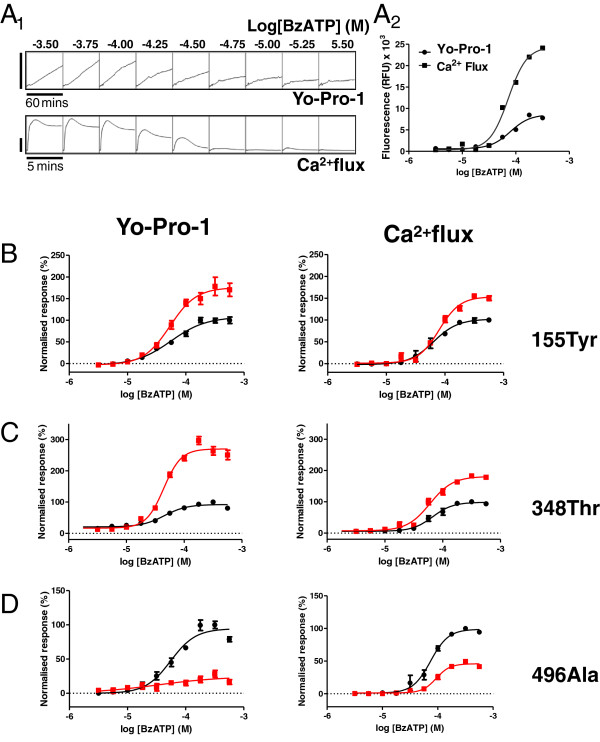
**Functional effects of different P2X**_**7 **_**SNPs. A**_**1**_. Concentration-dependent responses to BzATP on HEK-293 cells transfected with WT P2X_7_ cDNA. Representative example traces for the “pore” formation (top row, Yo-Pro-1 uptake) and the “channel” function (bottom row, Ca^2+^ flux) assays. Bar scales correspond to 10.000 RFUs. **A**_**2**_. Dose response curves for the same experiment shown in **A**_**1**_. **B**, **C** and **D**. Example CRCs obtained following application of BzATP for the 3 studied P2X_7_ SNPs (red symbols) normalized to the responses elicited by the WT variant (black symbols). Averaged data corresponding to multiple experiments are presented in Table 1.

Three previously described P2X_7_ receptor variants (SNPs rs208294 (His155Tyr), rs1718119 (Ala348Thr) and rs3751143 (Glu496Ala)) were analysed for their functional properties after transient transfection in HEK-293 cells. Pore function (Yo-Pro-1 uptake) and channel function (Ca^2+^ ion fluxes) were compared head-to-head by analysing concentration dependent changes induced by a standard P2X_7_ agonist (BzATP) by using FLIPR. Examples of agonist induced responses obtained in cells transfected with WT P2X_7_ cDNA following application of different concentrations of BzATP are shown in Figure 1A_1_ for Yo-Pro-1 uptake and calcium flux, respectively. Data from this representative experiment are also shown as dose response curves in Figure 1A_2_ yielding EC_50s_ of 78.82 μM and 72.58 μM, and Hill slopes of 2.37 and 2.5, for Yo-Pro-1 uptake and Ca^2+^ flux, respectively.

The data obtained with BzATP on the 155Tyr variant were similar to the WT in terms of both agonist potency (EC_50_) and Hill slope (Figure 1B and Table 1). However, significant differences in maximum responses were identified for both modes of activation (178 ± 6% and 126 ± 12% for pore and channel function, respectively, Table 1), confirming previous findings of a GOF phenotype driven by 155Tyr [22,23,25-27].

**Table 1:** **BzATP pharmacological parameters on the different P2X**_**7 **_**SNPs studied**

	**Yo-Pro-1 uptake**					**Ca**^**2+ **^**flux**				
	**WT**	**155Tyr**	**348Thr**	**496Ala**	**496Ala 155Tyr**	**WT**	**155Tyr**	**348Thr**	**496Ala**	**496Ala 155Tyr**
EC50 (uM)	46.64 ± 5.32	56.65 ± 2.05	45.24 ± 0.54	34.56 ± 5.12	53.18 ± 5.28	67.48 ± 2.1	71.04 ± 4.6	58.22 ± 1.54	96.66 ± 5.88 (*)	89.23 ± 3.45 (**)
Hill slope	1.27 ± 0.10	1.72 ± 0.04	1.77 ± 0.24	1.2 ± 0.51	2.01 ± 0.47	2.77 ± 0.25	2.52 ± 0.17	2.67 ± 0.42	2.67 ± 0.28	2.73 ± 0.34
Efficacy (%)	100	177.68 ± 6.18 (*)	218.06 ± 19.49 (*)	23.56 ± 2.5 (*)	33.46 ± 5.78 (*)	100	125.83 ± 11.74 (*)	137.23 ± 16.06 (*)	46.2 ± 6.87 (*)	54.14 ± 3.6 (*)
n	13	3	4	3	3	15	3	3	6	3

The EC_50_ and Hill slope values for the WT variant represent averages obtained from all data. The efficacy values are normalized to control experiments on the WT receptors evaluated at the same time. (*) = p < 0.001; (**) = p < 0.005.

The 348Thr variant was also characterised by similar BzATP EC_50_ and Hill slope to the WT, but, as with the 155Tyr variant, the maximum responses were dramatically increased to 218 ± 19% and 137 ± 16% for pore and channel function, respectively (Figure 1C and Table 1). While conflicting results are present in the literature for this specific SNP, our data are consistent with [23,27] showing that the 348Thr variant also generates a gain of function phenotype.

In striking contrast, the 496Ala variant, despite showing similar EC_50_ and Hill slope for BzATP, gave rise to significantly smaller signals in both the pore formation and channel assays. The maximal responses with this mutant were 24 ± 3 of WT and 46 ± 7% of WT for pore and channel function, respectively (Figure 1D and Table 1), confirming previous findings that this variant manifests itself with a LOF phenotype [24,28]. As previously reported [25], co-expression of the GOF SNP 155Tyr with the 496Ala LOF SNP in the same construct was not sufficient to rescue the LOF phenotype (Table 1).

### Effects of SNPs on P2X_7_ protein expression

**Figure 2:**
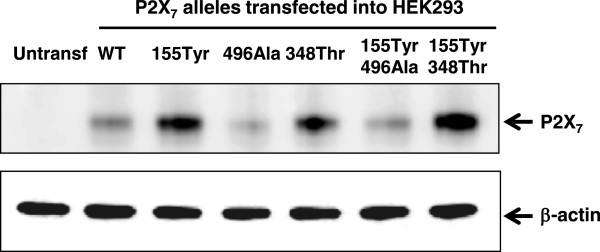
**P2X**_**7 **_**SNPs affect total protein levels in transfected HEK293 cells.** Total P2X_7_ protein levels were detected by Western blot using an anti- P2X_7_ antibody (Alomone). β-actin served as a loading control.

In order to better understand if the GOF and LOF phenotype could be driven by changes in protein expression, rather than changes in P2X_7_ function, we looked at P2X_7_ protein levels by western blotting. HEK-293 cells transfected with the various P2X_7_ cDNAs as described above for the functional studies, were detached, collected and homogenised. Western blotting was performed with a commercial C-terminus specific P2X_7_ antibody and positive bands at the expected size of ~65 KDa were clearly identified (Figure 2).

Despite utilising exactly the same amount of plasmids and exactly the same experimental and loading conditions, we found that the variant proteins were expressed at very different levels. Intriguingly, both the 155Tyr and 348Thr GOF mutants were expressed at higher levels, and the LOF mutant 496Ala at lower levels, than WT receptors, suggesting that a major functional effect of these SNPs is to modulate overall protein expression. Like in the functional studies described above, also at the protein level, co-expression of the GOF SNP 155Tyr with the 496Ala LOF SNP in the same construct was not sufficient to rescue the LOF phenotype.

### Sensitivity of P2X_7_ receptor variants to known P2X_7_ antagonists

**Figure 3:**
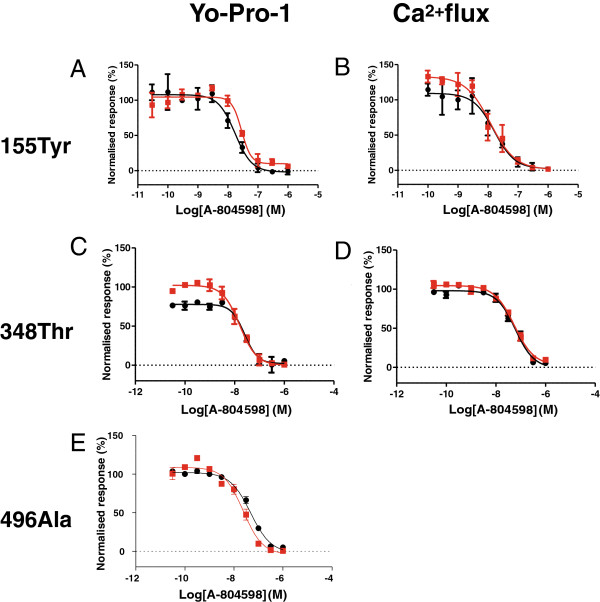
**A selective P2X**_**7 **_**antagonist shows similar activity on both WT and GOF variants.** Effects of A-804598 on agonist induced responses were studied in the FLIPR assay for both activation modes (Yo-Pro-1 uptake and Ca^2+^ flux). Data were normalized to the amplitude of responses elicited by the 100 μM BzATP. Only the results obtained with A-804598 in the WT variant (black symbols) and the 155Tyr **(A, B)** and 348Thr **(C, D)** variants (red symbols) are shown. Due to the low responses obtained with 496Ala in the Yo-Pro-1 uptake only data for the Ca^2+^ flux could be generated **(E)**. Averaged data for A-804598, as well as for the other two selective P2X_7_ antagonists used in this study, are presented in Table 2.

It is important for drug development to understand if these prevalent human P2X_7_ variants exhibit differential sensitivities to small molecule P2X_7_ antagonists that are under development to treat chronic pain conditions. We tested three different known P2X_7_ antagonists, A-804598 [29], A740003 [4] and AZ11654373 [30] (see Additional file 1) for their ability to block either channel or pore activity in the three P2X_7_ variants described above (Figure 3 and Table 2). We did not find any significant difference in these antagonists’ IC_50_ or Hill slopes for any of the P2X_7_ variants. Importantly, also the maximal block achieved was not different, for all the variants tested. Together with the results shown above of similar agonist sensitivity, these data suggest that despite different levels of expression, the various P2X_7_ variants might function, and be blocked, in a similar way.

**Table 2:** **Effects of selective P2X**_**7 **_**antagonists at GOF and LOF P2X**_**7**_**variants**

	**Yo-Pro-1 uptake (IC**_**50**_**, nM)**				**Ca**^**2+ **^**flux (IC**_**50**_**, nM)**			
	**WT**	**155Tyr**	**348Thr**	**496Ala**	**WT**	**155Tyr**	**348Thr**	**496Ala**
**A-804598**	21.77 ± 2.8	21.50 ± 5.8	15.5 ± 0.6	ND	20.81 ± 7.6	19.36 ± 6.9	40.7 ± 14.2	25.57 ± 1.6
**A740003**	332.83 ± 12.3	449.2 ± 12.7	415.9 ± 98.2	ND	507.87 ± 91.1	379.43 ± 135.1	720.75 ± 27.9	405.50 ± 81.7
**AZ11645373**	198.2 ± 42	342.6 ± 48.3	218.75 ± 113.5	ND	296.3 ± 98.5	510 ± 39.9	806.75 ± 249.3	412.40 ± 1.7

IC_50_ values reported here represent the averages ± SEM of 3 separate experiments. For simplicity Hill slope were not included in the table. Data for Yo-Pro-1 uptake assay for 496Ala variant could not be determined (ND) due to the low amplitude of responses obtained with this variant.

### Clinical studies

Demographic and baseline clinical characteristics of the DPNP patient population were collected at study randomization, ITT (intent to treat) and shown in Additional files 2 and 3. DNA samples were available from 159 Caucasian subjects. Most patients were males (59.8%). Most subjects had type 2 diabetes (89.9%) and were in their early 60s (average 62.6 years). The mean duration of diabetes was > 12 years and the mean HbA_1c_ was < 8%. The average duration of DPNP was 4.5 years [31]. The average BMI score was 35.1. The average daily pain severity score was reported at 5.9 ± 1.3. The average 24 hour worst pain score was 7.2 ± 1.4. The average pain score at night was 5.6 ± 2. Less than 5% of the DPNP patients had a comorbid major depressive disorder or generalized anxiety disorder. Pre-existing conditions included arthritis, osteoarthritis, and back pain, with frequencies between 10-15% [31].

The average scores from the MNSI are also provided to demonstrate that all patients reported neuropathic symptoms which were similar between male and female subjects.

The neuropathic symptoms most reported in the lower extremities were burning pain (91.1%), and prickling feelings (94.9%), but patients reported that they were able to sense their feet upon walking (78.8%). The physical assessment included the presence or absence of vibratory sense in the great toe, and the majority of patients (males = 98.9% and females = 92.2%) had decreased or absent vibratory sensation.

### *P2RX7* SNPs and pain severity in DPNP patients

**Figure 4:**
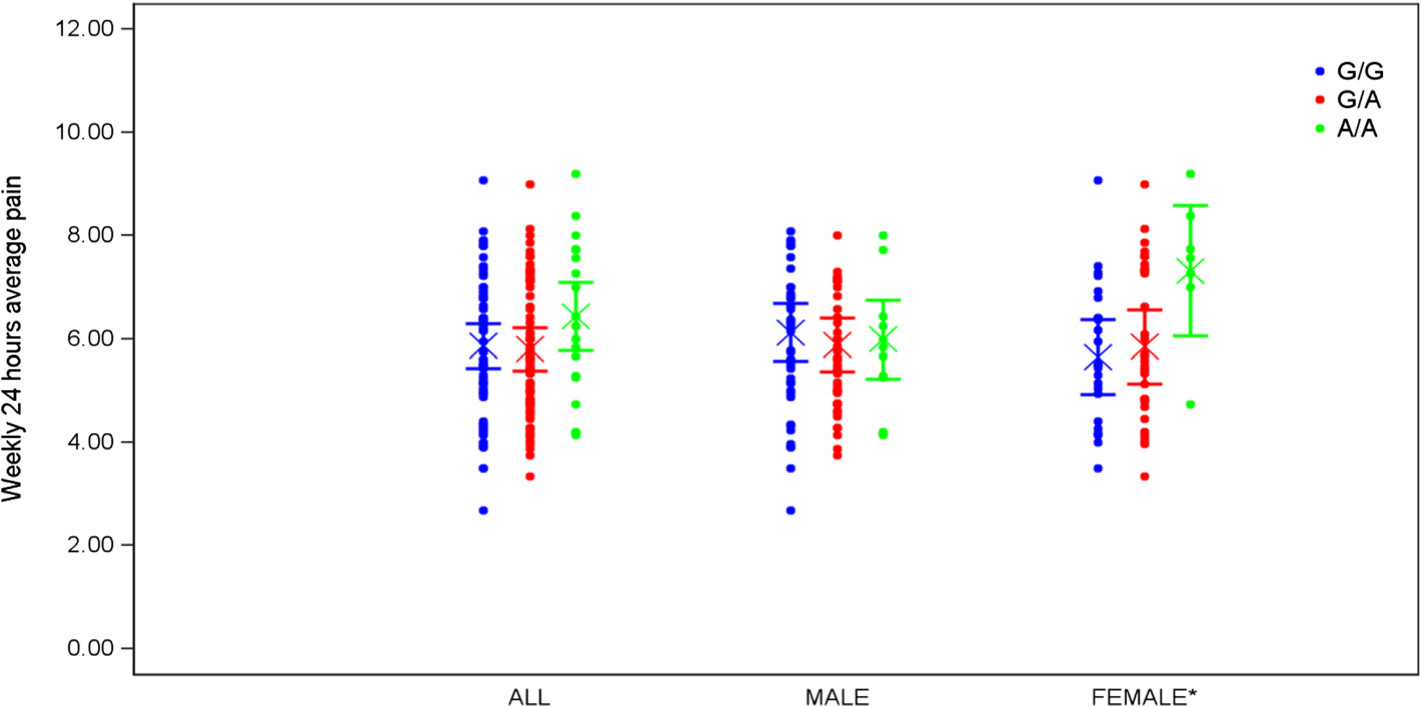
**Weekly 24 hour average pain intensity measured in patients with painful diabetic peripheral neuropathy (DPNP).** Measures were taken at baseline, using an 11 point Likert scale. These scores represent raw data and LS means and their 95% confidence intervals associated with the RS1718119 P2X_7_ SNP. Data are adjusted for baseline clinical characteristics in HMEZ ITT Caucasian patients and each gender subgroup. These data demonstrate that weekly 24 average hour pain intensity scores were significantly higher in females (p = 0.039).

Average pain severity scores of the DPNP patients were analysed in the context of the *P2RX7* SNP genotypes (Table 3). Using the additive model, none of the nine SNPs analysed achieved an un-corrected p-value < 0.05 in the ITT Caucasian population, when measured against the baseline score of weekly 24 hour average pain using the 11-point Likert scale. When the same analysis was split by gender, females carrying two copies of the A allele at the GOF SNP rs1718119 (Ala348Thr), had a 1.7 point covariate-adjusted higher mean baseline pain score than females carrying two copies of the G allele, as shown in Figure 4. Males showed no association with this SNP (un-corrected p = 0.54). A trend of increased baseline average pain score was also detected in female subjects with two copies of the T allele, versus two copies of the C allele at the GOF SNP rs208294 (His155Tyr) (T/T vs. C/C = 1.0 point covariate-adjusted mean baseline score, un-corrected p-value = 0.07, (see Additional file 4)).

**Table 3:** Weekly 24 hour average pain severity at baseline

			**All (N = 156)**		**Male (N = 94)**		**Female (N = 62)**	
**SNP**	**SNP ID**	**MAF**	**Mean effect**	**P-value**	**Mean effect**	**P-value**	**Mean effect**	**P-value**
1	rs208294	0.45	0.29	0.062	0.16	0.40	0.50	0.070
2	rs7958311	0.24	-0.10	0.57	-0.20	0.35	0.12	0.73
3	rs28360457	0.022	-0.78	0.13	-0.62	0.24	-2.26	0.15
4	rs1718119	0.36	0.18	0.26	-0.12	0.54	0.63	0.039
5	rs2230911	0.090	-0.12	0.66	-0.040	0.91	-0.35	0.43
6	rs10160951	0.003	0.18	0.90	0.26	0.84	-	-
7	rs2230912	0.13	0.16	0.50	-0.20	0.51	0.63	0.14
8	rs3751143	0.21	0.029	0.88	0.16	0.44	-0.17	0.71
9	rs1653624	0.013	0.38	0.59	0.41	0.53	-	-

Pain was measured by an 11-point Likert scale. Significance of association with *P2RX7* SNPs was evaluated under an additive model, adjusted for baseline clinical characteristics in non-Hispanic Caucasian subjects and by gender.

A trend of increased baseline average pain score was also detected in female subjects with two copies of the T allele at the GOF SNP rs208294 (His155Tyr) versus the other groups (1.3 point covariate-adjusted mean baseline score, un-corrected p-value = 0.004, (see Additional file 4)).

Sensitivity analyses were performed in the same genetic cohort using statistical models appropriate for recessive traits. Within those analyses, the effects of Ala348Thr and His155Tyr were still significant. The effect was strongest in the female cohort, and no effect was detected within males.

## Discussion

This study confirms that SNPs in the *P2RX7* gene are responsible for GOF and LOF phenotypes of the P2X_7_ receptor. Both channel and pore functions were affected in the same direction for all the SNPs studied. From a mechanistic point, the functional effects of the mutants on E_max_, and the results from the Western blotting assays, point to an increase in the number of receptors being expressed in the presence of the two GOF SNPs and a reduction in the presence of the LOF SNP. Additionally, our Western blotting assays demonstrated that when GOF and LOF SNPs were co-expressed in the same construct, the LOF effect was dominant. This may have relevance in some patients, as more than one SNP per *P2RX7* genotype has been described in humans [28]. Our results are different from earlier studies [22] but consistent with a recent publication [27] where both 155Tyr and 348Thr function and expression were studied using different techniques. Our results are very similar to those of Bradley et al. [27], with respect to 155Tyr, showing that an increased expression is driving the GOF phenotype, but not with respect to 348Thr. In our hands, like 155Tyr, 348Thr also drives more P2X_7_ protein to be expressed. Our own western blotting data are consistent with radioligand binding assays performed in our laboratory utilising the recently described radioligand [3H]A-804598 [29]. By executing binding assays on intact transfected cells, we indeed found a very similar trend in up- and down-regulation of P2X_7_ expression with the GOF and LOF variants (A. Mogg, preliminary data). Regardless of the exact cellular mechanisms underlying the process, both our data and those from [27] confirm that 348Thr also drives a GOF phenotype.

From a therapeutic point of view, understanding if these highly prevalent human P2X_7_ variants exhibit a differential sensitivity to small molecule P2X_7_ antagonists is critical. Profiling three known P2X_7_ antagonists, we did not identify any differences in their ability to block either the channel or pore functions of the variant receptors. The limited literature on this subject is still inconclusive, with one paper suggesting no changes with one specific P2X_7_ antagonist (A438079) tested at a single concentration [23] and a second paper suggesting a rightward shift in potency, albeit small, with a different P2X_7_ antagonist, GSK1370319A [32]. While the reported shift was not dramatic (~6 fold) it nevertheless highlights the absolute need to profile new and emerging drug candidates for their ability to differentially block P2X_7_ receptors in the wider human population. This preclinical translational work can raise confidence in the ability of new P2X_7_ directed drugs to be effective in the wider pain patient population or, alternatively, help in tailoring the treatment to the best responders and avoid treating non-responders.

Moreover, we provide new data from an additional independent pain cohort which support recent findings by other groups of a phenotypic relevance of distinct P2X_7_ GOF SNPs and extend previous findings on OA and PMP to a novel neuropathic condition, DPNP.

Two GOF SNPs rs1718119 (Ala348Thr) and rs208294 (His155Tyr), were nominally associated with a GOF pain phenotype in females but not males. Homozygous female patients with either of these SNPs had higher pain ratings across pain scales, consistent with other recently reported clinical pain conditions such as PMP and OA [21]. The same study [21] also identified a LOF SNP, Arg270His, associated with decreased pain that was not replicated in our study.

The association between genotype and pain sensitivity in the context of gender here described here for the GOF P2X_7_ SNP rs1718119 (Ala348Thr) are intriguing. A large body of evidence points to gender differences in the *prevalence* of pain disorders, with less robust data pointing to actual differences in pain *intensity* in a given disorder, as we are describing (for reviews see [33,34]). Earlier studies support our present evidence of a stronger correlation between particular SNPs and pain intensity in the female patient population, while the genetic-pain-gender links in additional relevant receptors and mediators are increasingly reported in the literature. For example, the melanocortin-1 gene has been shown to affect analgesic sensitivity in a gender-dependent manner [35] and SNPs in the mu-opiod receptor are associated with higher pain sensitivity in men but not women [36]. The issue of when these mutations occur and why they persist also merit consideration. Clinically, certain polymorphic variants of P2RX7 may identify patients, especially women, who are at greater risk of developing osteoporosis [37]. Some P2X_7_ SNPs eg Glu496Ala, have been associated with a higher prevalence of osteoporosis in Dutch women [38]. Conversely, other SNPs, eg *A348* were reported to be link to less bone resorption and potentially be protective against the development of osteoporosis, in Danish women [39,40]. At the preclinical level, P2X_7_ KO mice have been shown to express a gender-dependent phenotype when exocrine secretion [41] or bone remodelling [42] have been evaluated. In a transgenic model of ALS, mice have been shown to respond in a gender-dependent manner to a P2X_7_ blocker [43]. Overall, the previous studies and our present work support the idea that P2X_7_ polymorphisms play an important role in determining individual predisposition to pain sensitivity. More work is needed to understand the molecular and physiological nature and overall consequences of these associations.

While our study confirmed an association with increased pain sensitivity with the GOF Ala348Thr SNP as examined in McHugh et al. (2012), we did not detect a differential effect of GOF or LOF on a particular mode of P2X_7_ function, with both channel and pore activities being affected in the same way. We did not explore all possible SNPs, and our assay conditions could be slightly different from others. The findings in [21] suggested that a selective LOF of the pore function, and not the channel function, is linked to reduced pain sensitivity, based on mouse models. This may also be the case in humans, but still has to be demonstrated.

There seems to be good translation between results obtained studying recombinant P2X_7_ receptors expressed in heterologous expression systems and *in vivo* findings. Cellular receptor expression levels may impact the downstream pathways activated that consequently result in the increased or decreased pain phenotypes. Indeed, important mechanistic bridging studies at the cellular level have shown that gain of function P2X_7_ receptors are associated with an increased release of cytokines, like IL-1β, from human peripheral blood cells challenged with ATP analogues [23,44]. Increased IL-1β levels are reported in the CSF of patients suffering from different types of pain conditions, including neuropathies and chronic back pain [45]. Thus the P2X_7_ polymorphisms may act through neurogenic or inflammatory mediators to enhance or dampen the processes that converge and translate into pain sensitivities.

## Conclusions

Our results show that SNPs in the *P2RX7* gene receptor cause GOF or LOF of the P2X_7_ receptor and that “channel” function and “pore” function are affected in the same direction. Furthermore, the changes in function are associated with altered levels of protein expression. We also extend previous findings of an association between P2X_7_ polymorphisms and pain sensitivity in osteoarthritis and post-mastectomy pain patients, to a new population of diabetic painful neuropathic patients. We therefore believe that in the human population these SNPs may be relevant to the development and/or intensity of chronic pain states. Patients with some of these SNPs might benefit from new drugs specifically targeting these channels.

## Materials and methods

### Cloning of human P2RX7cDNAs

The wild-type *P2RX7* cDNA clone (Genbank accession: BC011913) was purchased from Openbiosystems (Fisher Scientific, Loughborough, UK) (Cat#: MHS1011-75778. Clone ID: 4298811). The various SNPs of *P2RX7* were generated by PCR-based mutagenesis using the wild-type cDNA clone as template. The nucleotide sequences encoding full-length wild-type and SNPs of *P2RX7* were inserted into pcDNA3.1(+) (Invitrogen, Paisley, UK) and verified by DNA sequencing. Two additional clones containing “double mutations” were produced where the 348 and 496 changes were added to the Tyr155 rather than to the “WT” His155 background.

### Transient transfection of P2X_7_ cDNA in HEK-293 cells

HEK-293 cells were cultured in high glucose DMEM medium (Invitrogen, Paisley, UK) containing 2 mM penicillin/streptomycin and glutamine and 5% BCS. Cells were plated at 9.6×10^6^ cells in a 75 cm^2^ flask, corresponding to ~80% confluence, and transfected after 24 h with 16 μg P2X_7_ plasmid by using Lipofectamine 2000 (Invitrogen, Paisley, UK) as a transfection reagent. 24 hours after transfection cells were detached using trypsin, counted and plated at a density of 5×10^4^ cells/well in 96-well black-walled FLIPR plates (BD Biosciences, Oxford, UK). Cells were kept in an incubator at 37°C and 5% CO_2_ and used for recording 24 hours after re-plating (i.e. 48 h after transfection).

### Functional analysis of P2X_7_ variants

#### Calcium flux assays (channel function)

Media was removed by inversion of the plate and 50 μl of buffer containing 4 μM Fluo-4 AM and 0.1% pluronic acid (Invitrogen, Paisley, UK) was added to each well. The assay buffer (HBSS, Invitrogen, Paisley, UK) contained (in mM): 1.3 CaCl_2_, 0.49 MgCl_2_, 0.41 MgSO_4_, 5.3 KCl, 0.44 KH_2_PO_4_, 4.2 NaHCO_3_, 138 NaCl, 0.34 Na_2_HPO_4_, HEPES 20 and pH adjusted to 7.2 (with NaOH). Plates were left in the dark for 1 hr at room temperature, Fluo-4 AM was removed by inversion of the plate, and the required volume of assay buffer was added to each well. Fluorometric Imaging Plate Reader (FLIPR, Molecular Devices, Sunnyvale, CA, USA) experiments were run at room temperature, and intracellular calcium levels were monitored before and after the addition of compounds. Differences between uptake values for the different mutants were analysed by T-test (GraphPad Prism).

#### Dye uptake assay (pore function)

A different assay buffer, lacking Ca^2+^ and Mg^2+^ was used for studying pore formation (DPBS, Invitrogen, Paisley, UK) containing (in mM): 2.7 KCl, 1.5 KH_2_PO_4_, 138 NaCl, 8.1 Na_2_HPO_4_ and 20 HEPES, pH adjusted to 7.2 (with NaOH). After removal of media cells were washed twice with assay buffer followed by addition of recording buffer containing 2 μM Yo-Pro-1 iodide (MW = 629, Invitrogen, Paisley, UK) a membrane impermeant nucleic acid dye used previously [46] for studying activation of P2X_7_ receptors. Changes in Yo-Pro-1 fluorescence were recorded at room temperature by using FLIPR for a total duration of 1 h following initial addition of test compounds. Concentration response curves for both calcium flux and dye uptake data were fitted to a 4-parameter logistic curve fit model using GraphPad Prism software. Differences between uptake values for the different mutants were analysed by T-test (GraphPad Prism).

### Western blots

Cells were harvested 24 h after transient transfection of P2X_7_-expressing plasmids in HEK-293 cells. Proteins were extracted using Cell Lysis Buffer (Cell Signaling Technology, Hitchin, UK), in the presence of protease inhibitor cocktail (Roche, Burges Hill, UK). Following centrifugation (400 g, 5 min, 4°C) to remove insoluble material, protein concentration was estimated by the Bradford method (Bio-Rad, Hemel Hempstead, UK), using bovine gamma globulin as the protein standard. Proteins (15 μg/lane) were prepared in 1x Laemmli buffer (Bio-Rad) and 50 mM DTT, heated at 37°C for 5 min, and resolved on a 4-12% Bis-TrisNu PAGE mini-gel (Invitrogen, Paisley, UK), transferred onto a nitrocellulose membrane (Bio-Rad, Hemel Hempstead, UK) and incubated in blocking solution 5% milk (Marvel, Sainsbury, UK) in TTBS (50 mM Tris-Cl, 150 mM NaCl, 0.1% Tween-20, pH 7.6 with NaOH) at 4°C, overnight. The membrane was cut at the 51 kDa MW marker, and the top part (>51 kDa) was incubated in blocking solution with anti-P2X_7_ antibodies (APR-004, Alomone labs, Jerusalem, Israel) at a 1:500 dilution, while the bottom part (<51 kDa) was incubated in blocking solution with anti-β-actin antibodies (Sigma-Aldrich Gillingham, UK) at a 1:10,000 dilution, for 1.5 h. Primary antibodies were detected with goat anti-rabbit (for P2X_7_) and goat anti-mouse (for β-actin) secondary antibodies conjugated to horseradish peroxidise (Sigma-Aldrich Gillingham, UK) at a 1:10,000 dilution, followed by signal development in West Femto reagent (Fisher Scientific, Loughborough, UK) and detection on an Image Quant Las 4000 Mini apparatus (GE Healthcare, Hatfield, UK). Beta-actin expression was used as a loading standard to confirm the quantitation of protein loaded per well.

### Patient population demographics and diagnosis

#### Sample collection and trial design

Blood samples were collected from the Phase 4 open label clinical trial study, “Duloxetine, Pregabalin, and Duloxetine Plus Gabapentin for Diabetic Peripheral neuropathic Pain Management in Patients with Inadequate Pain Response to Gabapentin: An Open-Label, Randomized, Non inferiority Comparison, (HMEZ).” The following criteria were used for inclusion in the trial: 1) Type 1 or Type 2 diabetes mellitus; 2) a glycated hemoglobin (HbA_1c_) level of ≤12%; 3) ≥18 years old; 4) DPNP as confirmed by a score ≥3 on section B of the Michigan Neuropathy Screening Instrument (MNSI) [47]; 5) daily pain intensity score ≥4, based on a numerical rating scale (0–10 points). Sample collection occurred within the trial period between 2006 and 2009. Blood samples were drawn at the baseline (randomization) visit, extracted for DNA, and stored at -80°C prior to assay. All information gathered and samples used were compliant with and approved by the institutional review boards of participating sites and all patients provided written informed consent [31].

### Patient instruments

MNSI: The MNSI is a validated instrument that assesses the presence and extent of neuropathy [47]. Part A is filled out by the patient and assesses the presence or absence of 15 items relating to pain and neurovascular insufficiency. Part B is filled out by the examining physician. It assesses the physical appearance of feet, and the additional presence, reduced signal, or absence of ulceration, reflexes, vibration, and sensation to monofilament application, for a total of 5 points per extremity.

Pain Intensity: was measured in patient diaries by the baseline (randomization) weekly mean of the daily 24-hour pain score (daily pain). Pain intensity was assessed using an eleven point numerical rating scale ranging from 0 to 10, depicting no pain to worst possible pain, respectively. Averages of worst pain and night pain ratings were two additional measures derived from the pain diaries that were also analysed.

### Genetic analysis of *P2RX7* gene polymorphism

One-hundred, fifty-nine self-reported non-Hispanic Caucasian subjects that met enrolment criteria for HMEZ were included in the genetic analysis. Eight non-synonymous SNPs (Table 3) were genotyped using Taqman allelic discrimination assays (SeqWright DNA Technology Services, Houston, TX). One SNP was assayed by Sanger sequencing at SeqWright DNA Technology Services due to Taqman assay failure. No template controls and four positive controls (Coriell CEPH) were run on each plate. Additionally, a subset of project samples was run in duplicate to measure concordance. A set of genotyping quality metrics were checked: per SNP call rate, per subject call rate, duplicate concordance rate and Fisher’s exact p-value for Hardy-Weinberg Equilibrium (HWE). For all subjects and across all genotypes, there was a 100% call rate. Assay duplicate concordance rate was 100%. All HWE p-values were non-significant (all p-values > 0.67).

### Statistical analysis

The clinical endpoints for the genetic association analysis included baseline pain severity measured by an 11-point Likert scale for scores of weekly 24 hour average pain, weekly 24 hour worst pain, and weekly average pain severity during the night. Three subjects did not have pain severity assessment measured. Each endpoint was analysed under an Analysis of Covariance (ANCOVA) model in the non-Hispanic Caucasian intent-to-treat (ITT) population from HMEZ who passed the genetic data quality control, as well as separated into male and female subgroups. Each *P2RX7* polymorphism was tested under an additive, recessive and dominant model for the minor allele. Besides the genetic effect, the ANCOVA had co-variate terms of HbA_1c_, diabetes type, BMI and age, and a term for gender when conducting analysis of both genders combined. Least square means by genotypic group were reported along with raw 2-sided p-values of genetic effect. In this exploratory setting, no multiplicity correction was applied.

## Abbreviations

ATP: Adenosine 5_-triphosphate; P2X_7_: P2X receptor subtype 7; P2RX7: Gene encoding P2X_7_; BzATP: 2_(3_)-*O*-(4-benzoylbenzoyl)adenosine 5_-triphosphate; Fluo-4: 2-{[2-(2-{5-[bis(carboxymethyl)amino]-2-methylphenoxy}ethoxy)-4-(2,7-difluoro-6-hydroxy-3-oxo-3*H*-xanthen-9-yl)phenyl](carboxymethyl)amino}acetic acid; MAF: Minor allele frequency; DPNP: Diabetic peripheral neuropathic pain; SNP: Single nucleotide polymorphism; GOF: Gain-of-function; LOF: Loss-of-function; OA: Osteoarthritis; PMP: Post mastectomy pain; BMI: Body mass index; MNSI: Michigan neuropathy screening instrument.

## Competing interests

All the authors are employed by Eli Lilly and Company, with the exception of WZ who is an employee of InVentiv Clinical Health.

## Authors’ contributions

DU and EL performed the in vitro functional studies on the P2X_7_ variants; CR, YWQ and KM contributed to the genetic identification of the P2X_7_ SNPs and the generation of the mutant cDNAs; PE, LM, BF and TAM performed the clinical and genetic analyses. AM performed the radioligand binding assays. OG performed the western blotting. ES supervised the studies and their planning, and drafted the manuscript. All authors read, edited and approved the final manuscript.

## Supplementary Material

Additional file 1 **Chemical structures of P2X**_**7**_** antagonists used in this study.** Click here for file

Additional file 2 Demographic and baseline characteristics for non-Hispanic Caucasian HMEZ ITT patients in the genetic study. Click here for file

Additional file 3 MNSI and secondary Likert pain scales. Click here for file

Additional file 4 **Weekly 24 hour average pain intensity measured by an 11 point Likert scale at Baseline, of patients with painful diabetic peripheral neuropathy (DPNP).** These scores represent raw data and LS means and their 95% confidence intervals associated with the RS208294 P2X_7_ SNP. Data are adjusted for Baseline clinical characteristics in HMEZ ITT Caucasian patients and each gender subgroup. These data demonstrate that weekly average 24 hour pain intensity scores were modestly higher in females (p = 0.070). Increased pain intensity scores were not appreciated. Click here for file
