# Supplementary figures and images for: Gain and loss of function of P2X7 receptors: mechanisms, pharmacology and relevance to diabetic neuropathic pain
Source: Mol Pain. 2014 Jun 16;10:37. doi: 10.1186/1744-8069-10-37 (PMC4072620; doi:10.1186/1744-8069-10-37)

## Slide 1
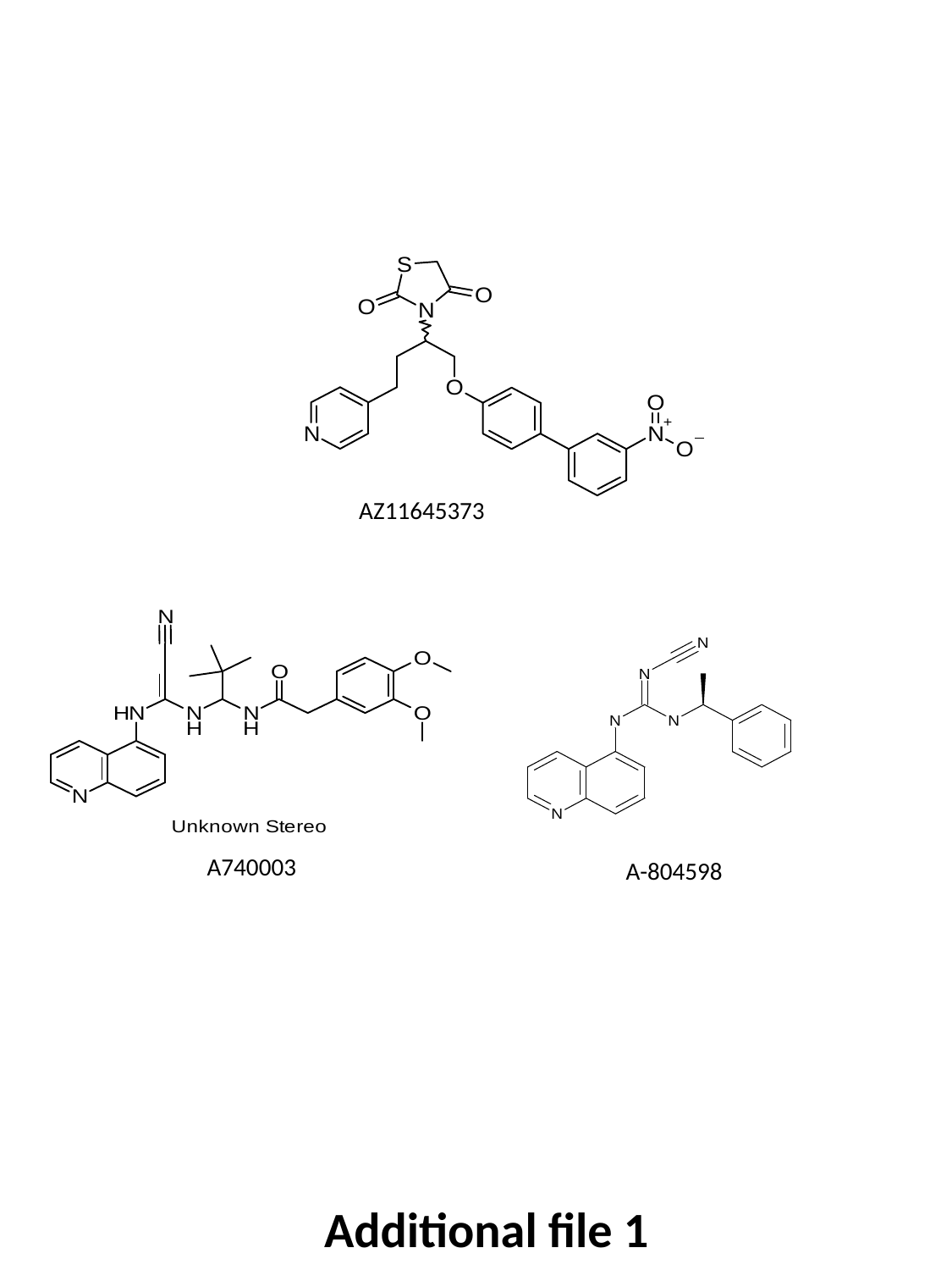

AZ11645373
A740003
A-804598
Additional file 1

Supplement: Additional file 1 — Chemical structures of P2X 7 antagonists used in this study. [file 1744-8069-10-37-S1.pptx]

## Slide 1
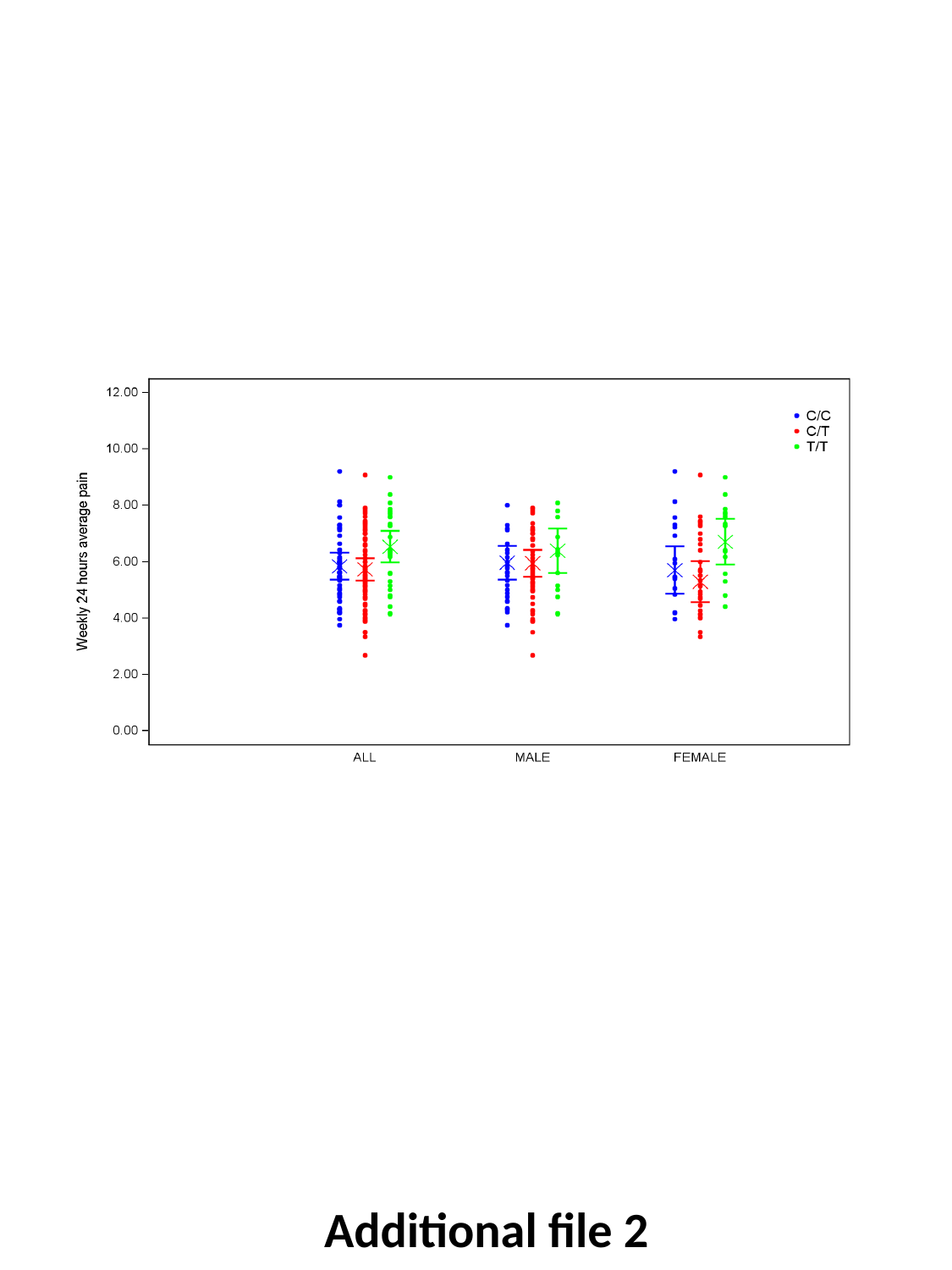

Additional file 2

Supplement: Additional file 2 — Demographic and baseline characteristics for non-Hispanic Caucasian HMEZ ITT patients in the genetic study. [file 1744-8069-10-37-S2.pptx]
