# Supplementary material for: Gain and loss of function of P2X7 receptors: mechanisms, pharmacology and relevance to diabetic neuropathic pain
Source: Mol Pain. 2014 Jun 16;10:37. doi: 10.1186/1744-8069-10-37 (PMC4072620; doi:10.1186/1744-8069-10-37)
Supplement: Additional file 3 — MNSI and secondary Likert pain scales. [file 1744-8069-10-37-S3.pptx]

## Slide 1
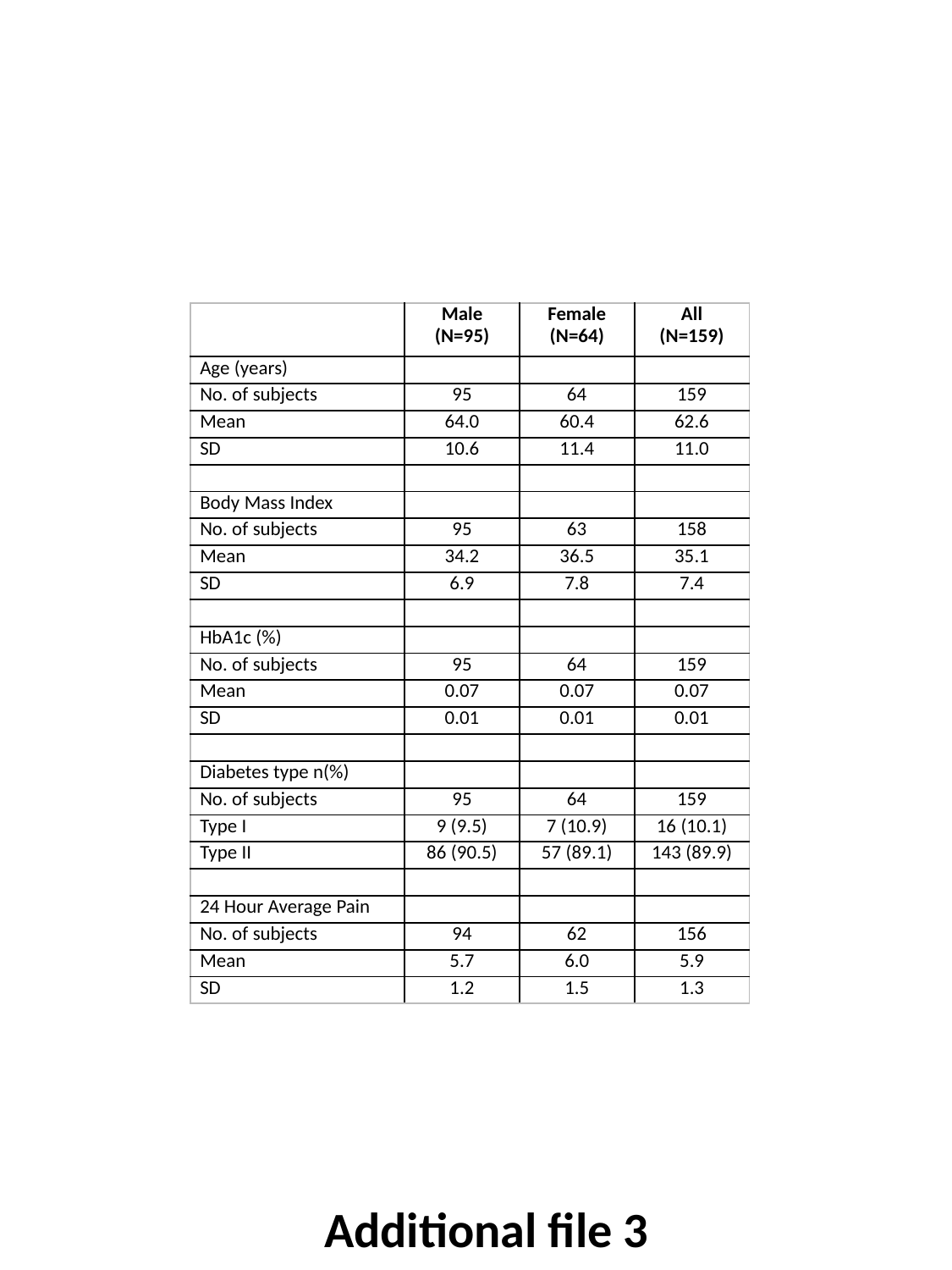

| | Male (N=95) | Female (N=64) | All (N=159) |
| --- | --- | --- | --- |
| Age (years) | | | |
| No. of subjects | 95 | 64 | 159 |
| Mean | 64.0 | 60.4 | 62.6 |
| SD | 10.6 | 11.4 | 11.0 |
| | | | |
| Body Mass Index | | | |
| No. of subjects | 95 | 63 | 158 |
| Mean | 34.2 | 36.5 | 35.1 |
| SD | 6.9 | 7.8 | 7.4 |
| | | | |
| HbA1c (%) | | | |
| No. of subjects | 95 | 64 | 159 |
| Mean | 0.07 | 0.07 | 0.07 |
| SD | 0.01 | 0.01 | 0.01 |
| | | | |
| Diabetes type n(%) | | | |
| No. of subjects | 95 | 64 | 159 |
| Type I | 9 (9.5) | 7 (10.9) | 16 (10.1) |
| Type II | 86 (90.5) | 57 (89.1) | 143 (89.9) |
| | | | |
| 24 Hour Average Pain | | | |
| No. of subjects | 94 | 62 | 156 |
| Mean | 5.7 | 6.0 | 5.9 |
| SD | 1.2 | 1.5 | 1.3 |
Additional file 3
