# Supplementary material for: Gain and loss of function of P2X7 receptors: mechanisms, pharmacology and relevance to diabetic neuropathic pain
Source: Mol Pain. 2014 Jun 16;10:37. doi: 10.1186/1744-8069-10-37 (PMC4072620; doi:10.1186/1744-8069-10-37)
Supplement: Additional file 4 — Weekly 24 hour average pain intensity measured by an 11 point Likert scale at Baseline, of patients with painful diabetic peripheral neuropathy (DPNP). These scores represent raw data and LS means and their 95% confidence intervals associated with the RS208294 P2X7 SNP. Data are adjusted for Baseline clinical characteristics in HMEZ ITT Caucasian patients and each gender subgroup. These data demonstrate that weekly average 24 hour pain intensity scores were modestly higher in females (p = 0.070). Increased pain intensity scores were not appreciated. [file 1744-8069-10-37-S4.pptx]

## Slide 1
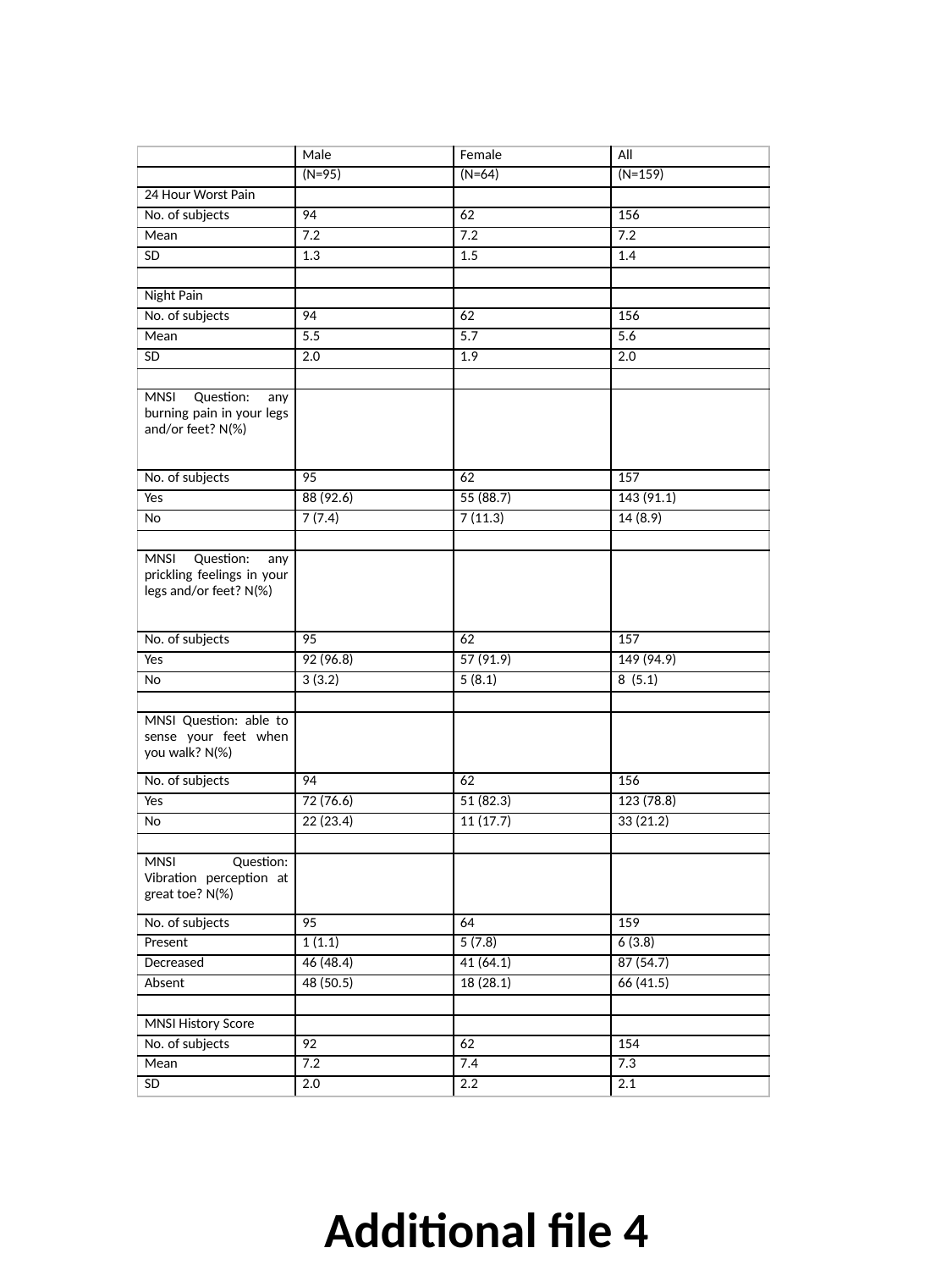

| | Male | Female | All |
| --- | --- | --- | --- |
| | (N=95) | (N=64) | (N=159) |
| 24 Hour Worst Pain | | | |
| No. of subjects | 94 | 62 | 156 |
| Mean | 7.2 | 7.2 | 7.2 |
| SD | 1.3 | 1.5 | 1.4 |
| | | | |
| Night Pain | | | |
| No. of subjects | 94 | 62 | 156 |
| Mean | 5.5 | 5.7 | 5.6 |
| SD | 2.0 | 1.9 | 2.0 |
| | | | |
| MNSI Question: any burning pain in your legs and/or feet? N(%) | | | |
| No. of subjects | 95 | 62 | 157 |
| Yes | 88 (92.6) | 55 (88.7) | 143 (91.1) |
| No | 7 (7.4) | 7 (11.3) | 14 (8.9) |
| | | | |
| MNSI Question: any prickling feelings in your legs and/or feet? N(%) | | | |
| No. of subjects | 95 | 62 | 157 |
| Yes | 92 (96.8) | 57 (91.9) | 149 (94.9) |
| No | 3 (3.2) | 5 (8.1) | 8 (5.1) |
| | | | |
| MNSI Question: able to sense your feet when you walk? N(%) | | | |
| No. of subjects | 94 | 62 | 156 |
| Yes | 72 (76.6) | 51 (82.3) | 123 (78.8) |
| No | 22 (23.4) | 11 (17.7) | 33 (21.2) |
| | | | |
| MNSI Question: Vibration perception at great toe? N(%) | | | |
| No. of subjects | 95 | 64 | 159 |
| Present | 1 (1.1) | 5 (7.8) | 6 (3.8) |
| Decreased | 46 (48.4) | 41 (64.1) | 87 (54.7) |
| Absent | 48 (50.5) | 18 (28.1) | 66 (41.5) |
| | | | |
| MNSI History Score | | | |
| No. of subjects | 92 | 62 | 154 |
| Mean | 7.2 | 7.4 | 7.3 |
| SD | 2.0 | 2.2 | 2.1 |
Additional file 4
